# The forgotten half: addressing the psychological challenges of wives of individuals with alcohol use disorder (AUD) in low- and middle-income countries

**DOI:** 10.3389/fpsyt.2024.1377394

**Published:** 2024-03-20

**Authors:** Akhil P. Joseph, Anithamol Babu, LT Om Prakash

**Affiliations:** ^1^ Department of Sociology & Social Work, Christ (Deemed to be University), Bangalore, Karnataka, India; ^2^ School of Social Work, Marian College Kuttikkanam Autonomous, Kuttikkanam, Kerala, India; ^3^ School of Social Work, Tata Institute of Social Sciences Guwahati Off-Campus Jalukbari, Assam, India

**Keywords:** wives of individuals with alcohol use disorder, low-and middle-income countries (LMICs), psychological challenges, mental healthcare, artificial intelligence-AI, machine learning (ML), virtual reality (VR)

## Introduction

The emotional and psychological challenges faced by wives of individuals with Alcohol Use Disorder (AUD) in Low- and Middle-Income Countries (LMICs) represent a crucial yet frequently overlooked aspect in the realms of social research and mental healthcare. These women, entrapped in a web of societal norms and stigmas surrounding AUD, endure their sufferings in relative obscurity (1). A meticulous keyword analysis of recent data from preeminent research databases, particularly the Web of Science, exposes a glaring deficiency in studies focused on understanding the experiences of these wives and their children (2). This research lacuna is not merely an academic oversight but mirrors a broader societal disengagement from the complex dynamics within families affected by AUD. Furthermore, cultural dimensions critically shape the experiences and responses of these women, who manage a challenging landscape marked by traditional gender roles, familial expectations, and societal perceptions of alcohol use and mental health. Gender-based expectations in many cultures within Low- and Middle-Income Countries (LMICs) compound this issue, as they may subject women to increased societal judgment and isolation. This significantly influences women’s perception of their experiences, their pursuit of support, and how their communities perceive them. The lack of focus on these cultural aspects leads to a superficial understanding of the nuanced impacts of AUD on these wives, who often silently shoulder complex, culturally influenced burdens, which make it crucial to address, particularly within contexts where resources are scarce and socio-cultural complexities add layers of challenges. This necessitates initiatives vital for enriching academic discourse and shaping healthcare practices that are empathetic and culturally attuned to the unique needs of these often invisible sufferers.

## Redefining support: bridging research and healthcare gaps

The experiences of wives of individuals with AUD in LMICs markedly differ from their counterparts in high-income countries (HICs), a disparity driven by socio-economic, cultural, and policy factors (3, 4). HICs typically offer greater availability of mental health services, including specialized treatments and robust support systems for families impacted by AUD, supported by extensive research and comprehensive data collection that facilitate targeted interventions. On the other hand, in LMICs, mental health services are often limited, underfunded, and disproportionately available in urban areas, leading to significant access challenges. The lack of resources and comprehensive data in these regions impairs the development of effective, context-specific interventions (5). Economic stability in HICs underpins stronger support structures at individual and policy levels, supported by a more robust healthcare infrastructure. This facilitates not only the provision of mental health services but also the implementation of policies and government support structures to address the ramifications of AUD on families. In contrast, LMICs struggle with economic constraints, under-resourced healthcare systems, and a paucity of supportive policies (6). Cultural and social attitudes further compound these challenges. HICs may exhibit greater openness and reduced stigma towards seeking help for AUD, while in many LMICs, cultural norms and stigmas often deter individuals from seeking help (7). These dynamics place a significant, frequently unrecognized burden on wives, who face societal stigma and the immense responsibility of caring for affected husbands without sufficient support or acknowledgement (8).

Acknowledging these distinct challenges is an urgent need for academic and healthcare institutions in LMICs to redirect their focus towards understanding and supporting the wives of individuals with AUD. This necessitates a robust academic inquiry, employing phenomenological, ethnographic, and longitudinal studies to probe into the psycho-social, economic, and cultural dynamics of wives having husbands with AUD. Such research should offer insights into their lived experiences, daily challenges, and evolving coping strategies in diverse economic and cultural contexts. Research tools must be culturally sensitive and linguistically adapted, including translations into various regional languages, to ensure inclusivity and relevance. Ethical considerations remain paramount, with strict adherence to confidentiality, anonymity, informed consent, and care to minimize risks of stigmatization and emotional impact. Moreover, research should refine mental health services to be highly responsive to the specific challenges faced by these women. This approach must be mindful of the limited resources and higher socio-economic pressures in these regions, contrasting with the more resource-abundant and socially supportive environments in HICs. Therefore, creating effective, feasible and sustainable strategies in resource-constrained settings while acknowledging the socio-economic, cultural, and structural differences is imperative.

## Technology-driven intervention strategies

The need to address the unique challenges and resource constraints prevalent in LMICs drives the choice to integrate technology-driven solutions like Artificial Intelligence (AI), Machine Learning (ML), and Virtual Reality (VR) into mental health interventions (9, 10). AI and ML offer innovative ways to develop predictive models for identifying at-risk women due to their husbands’ AUD, allowing for early and personalized interventions. These technologies can support the creation of AI-driven tools like chatbots for emotional support and online forums moderated for safety and relevance. Furthermore, AI and ML can analyze extensive data sets from varied sources, leading to the continuous refinement of services. VR therapies provide immersive experiences that can be particularly effective for stress management and emotional coping. The deployment of these technologies in LMICs is particularly significant, as they can bridge the gap caused by shortages of mental health professionals and other resources. They offer scalable, cost-effective solutions to reach a wider population, transcending geographical and socio-economic barriers.

While integrating technology-driven solutions presents immense potential, it also brings forth ethical considerations and concerns regarding the social divide (11). In LMICs, where digital literacy and access to technology can be limited, there is a risk of exacerbating existing inequalities. Ensuring equitable access to these technologies is paramount. Interventions must be designed with cultural sensitivity and inclusivity, acknowledging the diverse socio-economic backgrounds of the target population. Ethical deployment involves transparent data usage, protection of privacy, and consent protocols, ensuring that these technologies do not infringe on the rights or dignity of the individuals they aim to assist. Moreover, there is a need for continuous monitoring and evaluation to identify and mitigate any unintended consequences or biases that may arise from the use of these technologies. By addressing ethical concerns and reducing the social divide, we can effectively harness AI, ML, and VR to enhance mental health services in LMICs. This approach offers a more inclusive and equitable way to support the wives of individuals with AUD.

## Addressing the challenges in implementing the technology-driven AUD interventions

In addressing the implementation challenges of technology-driven solutions for AUD interventions in LMICs, a multi-pronged approach is essential to overcome barriers related to infrastructure, digital literacy, cultural nuances, and cost-effectiveness. Infrastructure challenges, such as limited internet connectivity and access to digital devices, can be mitigated by developing low-bandwidth and offline-capable AI and VR applications, ensuring functionality in settings with limited internet access. Developers could design these applications to download content during periods of connectivity for later use. Concurrently, addressing digital literacy is crucial; implementing training programs for healthcare workers and patients on using these technologies is key. Partnerships with local educational institutions or NGOs could facilitate digital literacy workshops, enhancing the adoption and effective use of these technologies. Moreover, embracing open-source AI and VR platforms can offer cost-effective solutions, potentially adopting a tiered service model where basic services are free or subsidized. To ensure cultural and linguistic appropriateness, developers should collaborate with local communities when developing AI models and VR content, integrating local languages and culturally relevant scenarios.

Expanding on integration strategies and aligning these technological interventions with national health policies is vital to ensure technology deployment is in tandem with governmental health goals and strategies. Integrating existing Electronic Health Records (EHR) systems is another crucial step, allowing AI and ML tools to enhance data analysis and patient management. Training modules for healthcare providers are essential for integrating these technologies into daily practice, focusing on ease of use and patient care benefits. Pilot programs in selected healthcare facilities can serve as initial testing grounds to refine the integration process. Further, the design of these technologies should be modular, allowing easy integration into existing healthcare systems without the need for extensive infrastructure changes. Integrating AI and VR tools with telemedicine platforms can be particularly beneficial for remote diagnosis and treatment in rural or underserved areas. Collaborative approaches are also fundamental, including forging partnerships with international tech firms, universities, and NGOs for technical expertise and funding. Local stakeholder engagement, involving local governments, community leaders, and patient advocacy groups, should meet community needs and preferences.

## Conclusion

The profound challenges wives face in LMICs, struggling with the repercussions of their husbands’ AUD, are exacerbated by a lack of targeted research and adequate support systems, presenting an urgent societal issue that calls for immediate, comprehensive intervention. This complex problem demands a collaborative, multidimensional response, necessitating an inclusive strategy combining rigorous research, establishing specialized mental health services, extensive public awareness campaigns, and adopting advanced technologies like AI and VR. Such a multidimensional approach is crucial to highlight the struggles of these wives, ensuring they receive the necessary attention, care, and support. Such a holistic perspective is vital for developing comprehensive family-centric interventions. By synergistically combining research, healthcare, and public advocacy, we can significantly lessen these wives’ burdens, empowering them to manage their lives better and surmount the difficulties posed by living with husbands suffering from AUD. This approach addresses the immediate needs of these wives and contributes to the larger goal of improving their mental health and overall well-being, thereby creating a more inclusive, supportive, and effective framework for families struggling with AUD.

## Author contributions

AJ: Conceptualization, Writing – original draft, Writing – review & editing. AB: Writing – original draft, Writing – review & editing, Conceptualization. LP: Writing – review & editing.
